# SARS-CoV-2 Survival on Surfaces and the Effect of UV-C Light

**DOI:** 10.3390/v13030408

**Published:** 2021-03-05

**Authors:** Anna Gidari, Samuele Sabbatini, Sabrina Bastianelli, Sara Pierucci, Chiara Busti, Desirée Bartolini, Anna Maria Stabile, Claudia Monari, Francesco Galli, Mario Rende, Gabriele Cruciani, Daniela Francisci

**Affiliations:** 1 Department of Medicine and Surgery, Clinic of Infectious Diseases, “Santa Maria della Misericordia” Hospital, University of Perugia, 06129 Perugia, Italy; sabrina.bastianelli@unipg.it (S.B.); sara.pierucci@unipg.it (S.P.); chiarabusti93@gmail.com (C.B.); Daniela.francisci@unipg.it (D.F.); 2 Department of Medicine and Surgery, Medical Microbiology Section, University of Perugia, 06129 Perugia, Italy; samuele.sabbatini@gmail.com (S.S.); claudia.monari@unipg.it (C.M.); 3 Department of Pharmaceutical Sciences, Unit of Nutrition and Clinical Biochemistry, University of Perugia, 06122 Perugia, Italy; desirex85@hotmail.it (D.B.); francesco.galli@unipg.it (F.G.); 4 Department of Medicine and Surgery, Unit of Human, Clinical and Forensic Anatomy, University of Perugia, 06129 Perugia, Italy; anna.stabile@unipg.it (A.M.S.); mario.rende@unipg.it (M.R.); 5 Department of Chemistry, Biology and Biotechnology, University of Perugia, 06123 Perugia, Italy; gabriele.cruciani@unipg.it

**Keywords:** COVID-19, SARS-CoV-2, UV-C, surfaces, persistence, glass, steel, plastic

## Abstract

The aim of this study was to establish the persistence of severe acute respiratory syndrome coronavirus 2 (SARS-CoV-2) on inanimate surfaces such as plastic, stainless steel, and glass during UV-C irradiation which is a physical means commonly utilized in sanitization procedures. The viral inactivation rate, virus half-life, and percentage of titer reduction after UV-C irradiation were assessed. Infectivity was maintained on plastic and glass until 120 h and on stainless steel until 72 h. The virus half-life was 5.3, 4.4, and 4.2 h on plastic, stainless steel, and glass, respectively. In all cases, titer decay was >99% after drop drying. UV-C irradiation efficiently reduced virus titer (99.99%), with doses ranging from 10.25 to 23.71 mJ/cm^2^. Plastic and stainless steel needed higher doses to achieve target reduction. The total inactivation of SARS-CoV-2 on glass was obtained with the lower dose applied. SARS-CoV-2 survival can be long lasting on inanimate surfaces. It is worth recommending efficient disinfection protocols as a measure of prevention of viral spread. UV-C can provide rapid, efficient and sustainable sanitization procedures of different materials and surfaces. The dosages and mode of irradiation are important parameters to consider in their implementation as an important means to fight the SARS-CoV-2 pandemic.

## 1. Introduction

Severe acute respiratory syndrome coronavirus 2 (SARS-CoV-2) is a coronavirus discovered in December 2019 in Wuhan City (the capital of Hubei province), China. It is the etiologic agent of the coronavirus disease 2019 (COVID-19) that was declared a global pandemic by the World Health Organization (WHO) in March 2020 [1].

SARS-CoV-2 is an enveloped RNA beta-coronavirus with a genome consisting of a non-segmented positive-sense, single-stranded ribonucleic acid (RNA). SARS-CoV-2 virions are spherical with a diameter ranging from about 60 to 140 nm and presenting the characteristic club-shaped spike (S, glycoprotein) projections from the surface. The surface is also formed by the envelope (E) and the membrane (M) glycoproteins. In the virion envelope, we found a helically symmetrical nucleocapsid (N) that contains the viral genome [2,3,4]. SARS-CoV-2 efficiently exploits the human angiotensin-converting enzyme 2 (ACE2) receptor for cells entry [4].

Respiratory droplets and aerosol particles exhaled during the speech of infected people have a crucial role in the spread of the infection [5,6]. Conversely, the role of inanimate surfaces in SARS-CoV-2 transmission is still controversial, as well as persistence on materials and stability outside the host. Several studies tested virus persistence on surfaces of common use such as plastic, glass, stainless steel, wood, paper, copper and cloth [7,8,9,10]. According to these studies, SARS-CoV-2 stability seems to be influenced by the characteristics of the different materials but also by environmental conditions such as temperature, pH and humidity [8,11,12,13].

In the light of the above, the efficient and rapid disinfection of surfaces and rooms may have an important role in the containment of the pandemic. In this context, beyond the standard disinfection methods such as chemical ones, ultraviolet germicidal irradiation (UVGI) has a potential role. In particular, UV-C light at wavelengths of about 254 nm exerts bactericidal and virucidal effects, and it is widely used in the environmental disinfection of enclosed spaces [14]. UV-C light induces chemical modifications of nucleic acids, leading to the blocking of replication mechanisms [15]. SARS-CoV-2 is highly susceptible to UV-C irradiation, and different studies demonstrated the rapid inactivation of the virus on surfaces and supplies [16,17,18,19,20].

The aim of this study was to establish the persistence of SARS-CoV-2 on inanimate surfaces such as plastic, glass, and stainless steel and the role of UV-C environmental disinfection as additional help in the fight against the pandemic. To achieve this, we performed virus survival tests to determine the physiological decay of virus infectivity under controlled conditions, and we assessed the effect of UV-C irradiation in an experimental model of fomites disinfection.

## 2. Materials and Methods

### 2.1. SARS-CoV-2 Strain and Vero E6 Cell Cultures

All experiments were performed using a SARS-CoV-2 strain isolated in our biosafety level-three (BSL3) virology laboratory in May 2020 from a symptomatic patient of Santa Maria della Misericordia Hospital, Perugia, Italy, as previously described [21]. Briefly, transport medium (UTM) of a nasopharyngeal swab was incubated with a 1:1 nystatin (10,000 U/mL) and penicillin–streptomycin (10,000 U/mL) mixture and was left to react for 1 h at 4 °C to remove bacterial/fungal contamination. The suspension was centrifuged at 400× *g* for 10 min, and the supernatant was inoculated on a Vero E6 cells monolayer maintained in Eagle’s minimum essential medium (MEM) supplemented with 10% fetal bovine serum (FBS) and 1% penicillin–streptomycin at 37 °C with 5% CO_2_. The supernatant viral titer was determined by the Median Tissue Culture Infectious Dose (TCID50) endpoint dilution assay [22] and stock aliquots were stored at −80 °C. The stock virus titer was 3.16 × 10^7^ TCID50/mL, and the frozen aliquots were thawed immediately before each experiment.

Whole-genome sequencing identified a SARS-CoV-2 genome belonging to clade 20A (lineage B.1), and clustered with viruses circulating in Italy in spring 2020 [23].

### 2.2. Inanimate Surfaces

The selected materials were plastic (polystyrene, 24-well plates, Corning, Falcon^®^, NY, USA), glass (12 mm diameters sterile disks), and stainless steel (AISI 304, 12 mm diameter sterile disks). Glass and stainless-steel disks were sterilized by autoclaving and laid on 24-well plates.

### 2.3. Virus Persistence on Inanimate Surfaces

All the experimentations were conducted in a BSL3 facility. Each experiment was conducted in triplicate and repeated at least thrice for each material. The temperature and relative humidity were monitored and maintained stable around 23–25 °C and 40–50%, respectively.

To determine the surfaces recovery efficiency, one 10 μL virus aliquot was directly processed for TCID50 determination, and another one was recovered by washings after 30 min of contact with surfaces (T_0_). The recovery efficiency was calculated as follows: [TCID50/mL recovered virus (T_0_) / TCID50/mL virus aliquot] × 100.

The virus persistence on surfaces was evaluated at different time points (0, 2, 6, 18, 24, 48, 72, 96 and 120 h). Vero E6 cells (3 × 10^3^ cells/well) were plated on 96-well plates 24 h before each time point.

Given the viral stock (10^7.5^ TCID50/mL), 10 μL were placed upon each material surface with a sterile pipet tip. The presumptive viral load (about 10^5.5^ TCID50) corresponds to that observed in the respiratory tract of COVID-19 patients [24]. Samples were recovered at time-points by washing surfaces twice with 500 μL of MEM. Each sample was diluted and inoculated in Vero E6 cell cultures for subsequent titer determination through the TCID50 endpoint dilution assay. To calculate the virus titer, the Reed and Muench formula [22] or Ramakrishnan formula [25], if the titer was too low to use the former, were used.

### 2.4. UV-C Irradiation Assay

As for SARS-CoV-2 persistence tests, 10 μL of frozen viral stock were placed on different surfaces of the materials by a sterile pipet tip.

A monochromatic UV-C (254 nm) lamp with a surface power density of 0.466 mW/cm^2^ was positioned 30 cm from the target. Starting from 10.25 mJ/cm^2^, corresponding to 21 s exposure, different doses of UV-C were applied until a titer reduction of 99.99% or above was reached. In parallel experiments, a polystyrene plate covered by aluminium foil was placed near the irradiated plate and used as a control (shielded plate).

After UV-C treatment, samples were recovered, as above described, and processed for TCID50 determination.

### 2.5. Statistical Analysis

Statistical analysis was performed using Graphpad Prism 8.3 (San Diego, CA, USA). The viral decay rate was estimated through linear regression, and the slope (with the respective standard deviation, SD, and 95% confidence intervals, CI) was used as an estimate of viral inactivation rate, expressed as TCID50/mL lost per hour (L_TCID50/mL_/h). Regarding the exponential decay, the virus half-life was computed as ln(2)/K (K is the rate constant, expressed in reciprocal of hours) with the respective 95% CI.

Data were tested for normality using the Kolmogorov–Smirnov test, and one-way ANOVA or Kruskal Wallis tests were applied as appropriate. Data were presented as the mean with the respective standard deviation.

## 3. Results

### 3.1. Virus Persistence on Inanimate Surfaces

SARS-CoV-2 persistence on inanimate surfaces showed differences among tested materials. The recovery efficiency from each material was calculated to consider a possible underestimation caused by the poor ability to recover all the viral particles placed in contact with the materials. The recovery efficiency was about 100 ± 14% for plastic, 87.9 ± 17.1% for stainless steel and 84.1 ± 22.5% for glass.

**Figure 1:**
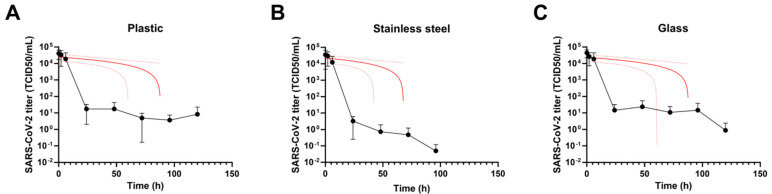
Severe acute respiratory syndrome coronavirus 2 (SARS-CoV-2) persistence on (**A**) plastic, (**B**) stainless steel and (**C**) glass. Ten microliters of SARS-CoV-2 viral stock were placed on different materials under controlled conditions (23–25°C and 40%–50% relative humidity). Infectious virus particles were carefully recovered at different time points (0 to 120 h) and processed for Median Tissue Culture Infectious Dose (TCID50) determination. SARS-CoV-2 infectivity kinetics (lines with filled dots) and linear regression analysis (red lines) are shown. Results are expressed as the mean ± standard deviation of 3 independent experiments (*n* = 3).

SARS-CoV-2 half-life on plastic was 5.3 h (95% CI 2 to 18.6 h), and the infectivity persisted until 120 h. The slope of the curve shown in Figure 1A is −279.7 L_TCID50/mL_/h (SD 82.8; 95% CI -541.3 to −108.1 L_TCID50/mL_/h).

On stainless steel, the virus maintained infectivity for a shorter period in respect to plastic. In particular, the virus half-life on stainless steel was 4.4 h (95% CI 1.3 to 25.7 h). Virus infectivity was detected on stainless steel no longer than 48–72 h, after which infectious SARS-CoV-2 was not recovered. The slope of the curve was −331.6 L_TCID50/mL_/h (SD 116.9; 95% CI −577 to −86.1 L_TCID50/mL_/h) (Figure 1B).

Concerning the viral kinetic on glass (Figure 1C), it showed a half-life of 4.2 h (95% CI 1.5 to 13.8 h), however, infectivity was detectable until 96–120 h. The slope of the curve was −268 L_TCID50/mL_/h (SD 77.8; 95% CI −429.5 to −106.6 L_TCID50/mL_/h).

Of note, in all the tested surfaces, the SARS-CoV-2 titer decrease was >99% after the drop drying, which took place starting from 6 h.

### 3.2. UV-C Irradiation Assay

Several experiments with different time-points and consequently different UV-C doses were performed for each material.

**Figure 2:**
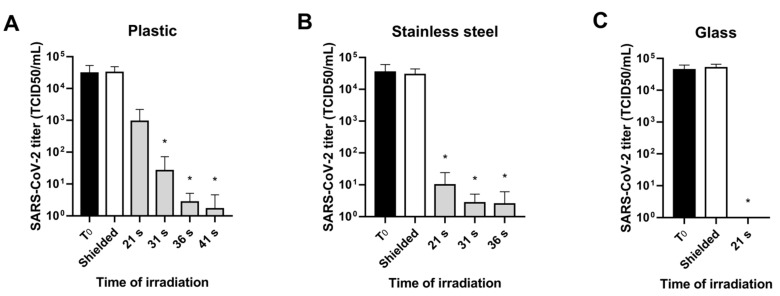
Effect of UV-C light (254 nm) on simulated fomites infection. Ten microliters of SARS-CoV-2 viral stock were placed on (**A**) plastic, (**B**) stainless steel and (**C**) glass, and recovered after 30 min (T_0_) or exposed to different doses of UV-C (grey bars). After treatments, infectious virus particles were carefully recovered by washings and processed for TCID50 determination. A polystyrene plate covered by aluminum foil served as the control (white bar “shielded”). Results are expressed as the mean ± standard deviation of 3 independent experiments (*n* = 3).* *p* < 0.05, UV-C treated material vs. UV-C shielded.

As shown in Figure 2, no significant differences were found between T_0_ and shielded plates and among shielded plates of different time-points (12–41 s).

The results were identical for all materials. UV-C doses tested for plastic were: 10.25 mJ/cm^2^ (21 s), 16.59 mJ/cm^2^ (31 s), 20.06 mJ/cm^2^ (36 s) and 23.71 mJ/cm^2^ (41 s). As shown in Figure 2A, the first dose (10.25 mJ/cm^2^) caused a titer reduction of about 2 logs. Using the dose of 16.59 mJ/cm^2^, we obtained a titer decrease of 3 logs. Finally, 20.06 and 23.71 mJ/cm^2^ broke down viral titer of 4 logs that correspond to a 99.99% reduction.

Similar results were obtained with stainless steel. The UV-C doses used were 10.25 mJ/cm^2^ (21 s), 16.59 mJ/cm^2^ (31 s) and 20.06 mJ/cm^2^ (36 s). As shown in Figure 2B, UV-C doses of 10.25 mJ/cm^2^ (21 s) and 16.59 mJ/cm^2^ (31 s) reduced viral titer of 3 logs, while the higher dose caused a titer reduction of 4 logs.

Differently, on glass, 10.25 mJ/cm^2^ (21 s) was a sufficient dose to eradicate SARS-CoV-2 (Figure 2C).

## 4. Discussion

The current pandemic of SARS-CoV-2 has deeply changed human social life and behavior. Following the already known and newest debated instructions of WHO [26], SARS-CoV-2 virus is easily transmitted between people by respiratory droplets and contact routes. The latter include surfaces in the immediate environment or with objects used by/on the infected people. Importantly, the environmental contamination of surfaces and objects cannot be ascribed only to symptomatic infected people since it has been demonstrated that the viral load is likely to be the same as asymptomatic ones [27,28]. These data force one to keep disinfection practices active and constant even in environments not only frequented by confirmed patients. SARS-CoV-2 transmission via speech-generated respiratory droplets is responsible for most of the viral transmission and the environmental disinfection without ventilation improvement is not enough to reduce the viral spread in indoor spaces [29]. In fact, another important route of virus transmission is aerosolization during human speech or normal breathing [30]. In this setting, SARS-CoV-2 particles emitted in aerosols could remain viable for hours and be carried over longer distances. Furthermore, the drying of droplets could generate aerosol-sized particles able to remain suspended, but the half-life is, however, much shorter than on hard surfaces.

The attention paid to hygiene practices towards the body and the surfaces has led to several modifications in human habits and to the development of innovative and efficient cleaning procedures. As well as the chemical ones, physical disinfection methods may be rapid and less time-consuming thanks to the possibility for them to be exerted by automated instruments and to the possibility of being carried out on solid surfaces and aerosols [14].

The contact transmission of SARS-CoV-2 has rapidly gained attention after researchers found the long persistence of infectious viral particles on a plethora of materials [7,31,32,33]. Even if the comparison between the different experimental conditions is quite hard to be correctly carried out, several studies share the belief that the survival of this new coronavirus ranges between hours to weeks. The different starting inoculum size, ambient conditions, materials and the evaluation and quantification of the viral titers can cause discrepancies in giving a unique description of the physiological decay phenomenon of the virus outside the host. It is necessary to premise that no experimental system would be able to faithfully reproduce reality, since it has been shown that the size of virus-laden droplet emitted by infected people should be around 10–20 µm [34,35]. In this regard, in our experimental conditions we used a virus inoculum of 10 µL that is a compromise between other researchers’ approaches with 5 to 50 µL volumes. The reason behind the choice would be to include any more abundant secretions that could be accidentally released, such as saliva and mucus.

Our results confirmed the ability of SARS-CoV-2 to persist on most common materials such as stainless steel, plastic, and glass with a half-life of 4.4, 5.3 and 4.2 h, respectively. We selected these materials because it has been recently demonstrated that the SARS-CoV-2 spike protein shows the highest affinity for polystyrene, followed by stainless steel and glass [36].

In all the tested materials, we observed a drastic titer decrease between 6 and 24 h after the deposition. We demonstrated that the decrease was concomitant to the drying of the droplet because most of the droplets were found to be dry at the time of 6-h analysis, and this phenomenon can be explained by recent findings. In fact, Corpet D.E. suggested that dryness could inactivate enveloped viruses such as SARS-CoV-2 [37], but, at the same time, this virus is able to remain still infectious on surfaces after drying [6,38,39]. Furthermore, performing an additional experiment by incubating the same virus concentration in 1 mL at 37 °C for 24 h did not show the same decrease in viral titer after TCID50 determination, but it was about 50% (data not shown), confirming that drying is decisive in the observed titer decrease. The longer persistence that has been obtained in other studies is sometimes linked to a high inoculum size used (e.g., 50 µL) that clearly takes more time to dry in respect to our conditions. In addition, as stated above, even a smaller inoculum has been shown to be more persistent in some papers [8], so all these considerations strengthen the statement that there is not a standardized method for this kind of experiment. We have to highlight that our experimental endpoint was 120 h after inoculum laying because the time to complete extinction of virus infectivity was not our aim.

After all, it is still unknown the minimum viral load that may lead to the disease onset by the contact with an infected surface and the subsequent transport to the entrance mucous membranes, but it has been shown that contact transmission varies according to the characteristics of materials [40]. Indeed, the novelty of our paper was to test the effect of UV-C treatment on viral particles laid onto different materials since there are some papers describing the direct effect without considering the influence of the substrate. It has been reported that single-stranded RNA viruses can be highly affected by UV-C irradiation and that the effect could vary with the type of surface [41]. To the best of our knowledge, this is the first paper on SARS-CoV-2 describing the effect of UV-C on different infected materials together with the physiological viral infectivity decay as well. As for virus survival tests, other studies previously evaluated the effect of UV-C light on SARS-CoV-2 inactivation by using different experimental conditions often consisting in higher virus inoculums (50 to 600 µL) [16,17,18,42,43] in respect to our approach that should be closer to possible scenarios of surfaces contamination. Indeed, the UV-C doses found to reduce the viral titer of about 3 logs (99.9% reduction) were much higher than ours. Based on our results, a smaller dose of UV-C (10.25–23.71 mJ/cm^2^) is enough to reduce the viral titer of >99.99%. In particular, plastic seems to be the most refractory material to UV-C disinfection, followed by stainless steel and glass, where the latter showed the best compliance with the treatment. In light of the above, we can conclude that UV-C irradiation could be a quick and effective means for the disinfection of indoor spaces, and these new data could be helpful in the setting of irradiation-based sterilization.

## 5. Conclusions

The developments of the epidemiological situation have demonstrated that social distancing, the application of personal protective equipment, and all the restricting and limiting measures imposed on the population have just postponed multiple waves of SARS-CoV-2 infections worldwide.

All existing data, together with our findings, suggest the crucial role of a rapid and efficient disinfection of indoor environments to counteract the spread of highly infective agents such as SARS-CoV-2. In particular, these practices should be implemented in a certain time frame: the period in which the viral particles are able to remain infectious and which probably could maintain a sufficient titer to be considered a true source of viral transmission. Moreover, the disinfecting efficacy of UV-C light found to be material-dependent focuses on the choice of materials to be preferred in constantly frequented environments. 

## Data Availability

The datasets used and/or analyzed during the current study are available from the corresponding author on reasonable request.
